# The Financial Crisis at Worcester Infirmary

**Published:** 1913-08-09

**Authors:** 


### THE FINANCIAL CRISIS AT WORCESTER INFIRMARY.

### Its Future as a Training School.

As intimated in last week's Hospital, a special meeting of governors was held on the 29th ult. to hear the report of the Executive Committee on the financial crisis and their proposals for dealing with it. The Bishop of Worcester presided, and twenty-nine governors were present. In opening the proceedings, the Bishop expressed deep regret at contemplating any curtailment of the work, but they were there as business people. Two years ago he had felt obliged as President to call the governors' attention to the fact that they were very largely exceeding their income, and, in fact, were living upon the expectation of legacies. Then the Insurance Act was passed, and consequently subscriptions fell off. A great number of large London hospitals might weather the storms ahead, but the smaller town hospitals would be in a very perilous position before long. It was their wisdom to be businesslike now and to harden their hearts in the hope that the public would really see what was happening, and by doing that they would be very likely to produce a great revival of subscribers. The Secretary then read a statement of the crisis, which has already been reported.

Its adoption was moved by the Chairman of Committee, Mr. G. Shrewsbury Smith, who said they could not do more to bring home to the public the needs of the infirmary. They had critics and opponents; some declined to help because they were selling out stock, others declined to subscribe as long as they had invested capital. Some people had said they were extravagant, but that suggestion he strongly repudiated, for, comparing the Worcester Infirmary with 57 provincial hospitals, the charge per bed was £5 7s. less than those elsewhere. He hoped the people of Worcester would feel it a stigma upon their character to allow the infirmary to close down so many of its beds, and hoped the necessary money would soon be forthcoming to enable them to re-open them. The resolution was seconded by Mr. Park, but opposed by Mr. Sydney Harris, who proposed an amendment: "That the question be referred back to the committee to devise means to bring up the receipts more into line with the expenditure." He said that if the proposals were carried, they would deprive 500 sick poor in the city and neighbourhood of benefits. [This figure is based on the calculation that the 130 beds are always occupied.] He suggested that it would help the committee materially if someone would take over their investment in corporation stock, for they lost 10 per cent. on selling it. The committee still had £14,000 funded property which they could draw upon (even assuming no legacies came in), and it would carry them on for another five years. He contended that the committee's proposals were not businesslike, because if the number of beds was reduced the infirmary would cease to be a training school. Mr. Gostling said it would not be recognised by the Army and Navy, that was all. Mr. Harris said the result would be that they would get a different standard of nurse and sister. He hoped that, until the time came for the State to support the infirmary, they would carry on the work in the same manner as they had done hitherto.

Mr. Boyce, in seconding, said he had examined the figures and he found that the cost per bed had increased 50 per cent. during the last 20 years, and that salaries had increased 100 per cent. He contended that the increase was greater than was experienced in business concerns. He advocated personal application to people in a position to become subscribers. Mr. Gostling (senior surgeon) remarked, in reference to the statement as to the increase in the cost per bed and in salaries, upon the great improvement which had been made in the service given to patients. Whether the infirmary would be a good training school with fewer beds would depend on the nature of the cases they treated. If they took the severer cases, and kept up the quality of the sisters by paying well, they could train as well with the small number of beds as with the larger. Whether they should ask the governors to keep up a big hospital to train nurses was another matter. The Hon. Mrs. Britten said an anonymous donor had offered, through her, to give £100 if 24 others would give or collect £100 each, provided the infirmary were carried on as at present. She thought the institution might appeal more to the county if it were called "The Worcester County Hospital."

Canon Longhurst thought that between those who now went to the hospitals for free treatment and those who could afford to go to a nursing home there were a vast number who could afford from £3 3s. to £5 5s., and if the committee did not do something to try and make a half-way house for these people, he thought they would be failing in their duty.

The amendment was lost, and the recommendations largely to reduce the number of beds were adopted by seventeen votes to seven.

